# Siamese Tracking from Single Point Initialization

**DOI:** 10.3390/s19030514

**Published:** 2019-01-26

**Authors:** Zheng Xu, Haibo Luo, Bin Hui, Zheng Chang

**Affiliations:** 1 Shenyang Institute of Automation, Chinese Academy of Sciences, Shenyang 110016, China; luohb@sia.cn (H.L.); huibin@sia.cn (B.H.); changzheng@sia.cn (Z.C.); 2 Institutes for Robotics and Intelligent Manufacturing, Chinese Academy of Sciences, Shenyang 110016, China; 3 University of Chinese Academy of Sciences, Beijing 100049, China; 4 Key Laboratory of Opto-Electronic Information Processing, Chinese Academy of Science, Shenyang 110016, China; 5 The Key Lab of Image Understanding and Computer Vision, Shenyang 110016, China

**Keywords:** object tracking, contour detection, Siamese network, deep learning

## Abstract

Recently, we have been concerned with locating and tracking vehicles in aerial videos. Vehicles in aerial videos usually have small sizes due to use of cameras from a remote distance. However, most of the current methods use a fixed bounding box region as the input of tracking. For the purpose of target locating and tracking in our system, detecting the contour of the target is utilized and can help with improving the accuracy of target tracking, because a shape-adaptive template segmented by object contour contains the most useful information and the least background for object tracking. In this paper, we propose a new start-up of tracking by clicking on the target, and implement the whole tracking process by modifying and combining a contour detection network and a fully convolutional Siamese tracking network. The experimental results show that our algorithm has significantly improved tracking accuracy compared to the state-of-the-art regarding vehicle images in both OTB100 and DARPA datasets. We propose utilizing our method in real time tracking and guidance systems.

## 1. Introduction

Visual object tracking is fundamental in various tasks of computer vision, such as video surveillance [1], augmented reality, or autonomous and assistance systems, such as automatic driving [2]. This paper focuses on the problem of visual object tracking, which is one of the most active research areas. Tracking generally describes the task of detecting and following one or more than one objects in a video sequence, where substantial strategies are used. For instance, tracking by simple template, tracking by salient image feature, or tracking by highly adaptive online learning.

A large amount of previous work has been done in both single object tracking [3,4,5,6,7] and multiple-object tracking [8,9,10,11,12,13,14]. There are different kinds of challenges in object tracking, such as appearance variance caused by motion, illumination, occlusion, and deformation [15,16]. Tracking by detection [17,18,19,20,21,22] is one of the normal strategies to deal with these challenges. However, most of them depend on bounding boxes which are a four-dimensional vector with x, y coordinates, width, and height as input for target representations, and typically suffer from the problem of bounding boxes drifting. The drifting problem may be caused by several reasons: target occlusion, articulated or non-rigid motions, confusion of foreground and background, etc. Take confusion of foreground and background for an example, with only a bounding box region in the first frame as the known target, it is difficult to differentiate the target from the cluttered background especially when the target itself moves, or has a change of appearance.

Recently, tracking algorithms based on segmentation have been investigated [23,24,25,26,27], but only pixel-level information is utilized in semantic structure modeling for targets in these methods. If higher level semantic information is required, that will introduce a huge amount of computation. Most methods extract semantic information depending on deep convolutional neural networks (CNNs) and are not able to achieve real time requirement. On one hand, real-time tracking is demanded in most of the applications. On the other hand, many approaches also adopt online training, which is computationally expensive, to boost the performance. Conventionally, it is difficult to satisfy both real time and high performance in a single tracker. Therefore, it is very crucial to balance the tracking performance and the time consumption. 

As mentioned above, both traditional bounding boxes methods and pixel-level tracking by segmentation methods have their respective drawbacks. In our task, our mission is to locate and track vehicles in real time. These vehicles have small size in images. As a result, it is difficult for a human to locate vehicles precisely in real-time video. Another problem is that taking fixed bounding boxes as input in most tracking algorithms is not suitable for our condition, because too much cluttered or even deceptive background information makes the small size target unable to be differentiated. We combined the ideas from both of the two and propose a new method that may be effective to model both feature-level and semantic-level information of the target. The performance degradation by the reduction of online training or learning can be offset to a certain extent with this reliable and information-rich target template. By avoiding time-consuming online training, offline training is adopted in our system instead. While GOTURN [28] regards object tracking as a box regression problem, we use a Siamese network to regard it as a similarity learning problem. Finally, we implement our system with high performance with relatively high speed in real time.

Our contributions can be concluded as follows: (1) We propose and implement the function of a target selection module by “start with a point” which has advantages over the traditional bounding boxes for start-up in tracking methods; (2) We modify and combine the contour detection network and the fully-convolutional Siamese tracking network to track in real time using shape-adaptive templates; (3) It achieves leading performance for vehicle videos in both OTB100 and Defense Advanced Research Projects Agency (DARPA) Video Verification of Identity (VIVID) datasets.

The rest of the paper is organized as follows. In Section 2, we introduce the related research work about system design, object contour detection proposal, and Siamese networks. In addition, we show the example of failed tracking by Siamese network with bounding box input due to the interference of background information. In Section 3, we describe the details of our approach to the contour detection network architecture, the template extraction module, the fully convolutional Siamese network architecture, and the details of the end-to-end training. Experimental results on the OTB100 dataset and DARPA datasets are showed in Section 4. Section 5 concludes the paper.

## 2. Related Work

In this part, we will introduce our related work from three aspects: (1) Algorithm Design, (2) Bounding Box Proposal vs. Object Contour Proposal, and (3) Siamese Network.

### 2.1. Algorithm Design

**Figure 1:**
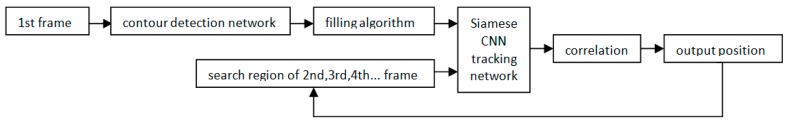
Block diagram of the main steps.

Unlike common start-up with an initial bounding box in most of the tracking process, our requirement is to select a target in real-time video by clicking on it on the monitor screen. Upon clicking on any part of the object, the tracking information is learned automatically by the system. To be specific, our system will detect and segment the contour of the selected target on a semantic level. After that, target feature can be extracted depending on the contour model without background information. In other words, we let the tracker know which object to track rather than a bounding box region. Regarding the contour detection network, we adopt and modify the VGG-16 network [29], which performs the state-of-art ImageNet challenge [30]. In detail, we adopt the front-end which has done well in extracting features and modify the back-end to make the network available to extract contour information. Using the shape-adaptive segmented template as input instead of a bounding box region successfully solves the problem of bounding box drifting regarding complex or deceptive background. Then, a filling algorithm is utilized on the output of previous contour detection network to create a template and start the tracker. Now, we take the template and the search region of each frame as inputs of the Siamese tracking network which generates a maximum correlation to denote new output position. A block diagram of the main steps of the whole process is shown in Figure 1.

### 2.2. Bounding Box Proposal vs. Object Contour Proposal

Visual object detection and tracking are two of the most fundamental and challenging computer vision problems. They are highly interrelated in most of the tasks, detection provides input that can guide tracking and enhance its performance. At the same time, an accurate solution to the detection problem provides reliable observations to tracking.

There are various object detection methods with outputs of bounding boxes. Most state-of-art algorithms are mentioned in both Pont-Tuset et al. and Hosang et al.’s work [31,32]. These methods are motivated by the requirements of target detecting or tracking and widely used in many applications. However, compared with object contour detection, the drawback of these bounding boxes is that their results are not accurate enough to locate the objects. By contrast, the results in object contour detection clearly show the shape of the target and its exact location. Instead of bounding boxes, using target contour for detecting or tracking purpose cannot only help with improving the accuracy, but can also tell the system what the target might be according to the shape. 

**Figure 2:**
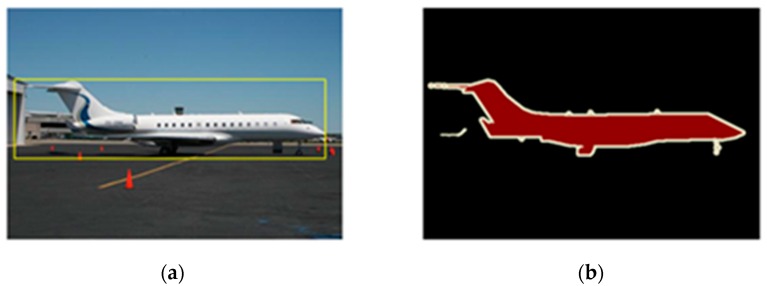
Results of both bounding box proposal method and object contour proposal method: (**a**) Bounding box proposal; (**b**) Object contour proposal.

Figure 2 shows an example of the results in both bounding box proposal method and object contour proposal method: the result of a bounding box proposal method is a coarse window, while the result of an object contours proposal method are fine pixels. Compared with the result in the bounding box method, the result of the object contour method contains most of the target information and the least background information. From the bounding box in the image on the left-hand side, the sky, the landing ground, and the buildings which belong to background information are also taken into account as target information during the feature extraction process in tracking. 

### 2.3. Siamese Network

Recently, Siamese networks have been widely utilized in many visual tracking tasks. A Siamese network has two branches; each of them encodes different features into another representation domain and then compares with each other by fusing with a specific tensor. Finally, a single output is produced to display results such as similarity. Siamese networks have balanced accuracy and speed because they transfer the traditional visual tracking from searching tasks to comparison tasks.

**Figure 3:**
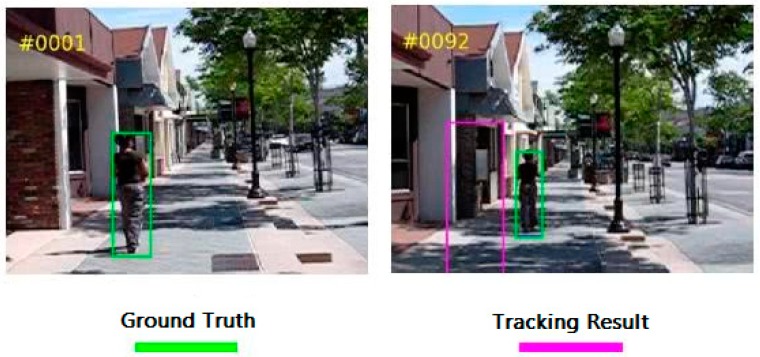
An example of false tracking due to target and background mixing: the green box indicates ground truth and the purple one indicates tracking result.

For instance, GOTURN is one of the networks using Siamese network in feature extraction process and adopts fully connected layers as its fusion tensor. In this regression method, the last bounding box is used to predict a current one. The real-time recurrent network (Re3) [33] also employs Siamese network, besides, a recurrent network is adopted to make the template branch to produce better feature. Siamese-FC [34] outperforms GOTURN and this owes to three reasons: the first reason is that its fully convolutional network architecture makes the offline training data highly discriminative; the second reason is that Siamese-FC adopts a densely supervised heat map instead of one proposal regression in GOTURN; last but not least, a correlation layer is utilized as a fusion tensor to improve the accuracy, which is attributed to the success of those correlation based methods on visual object tracking. Although a lot of latest discriminative correlation filter (DCF) methods, such as LADCF [35] and MFT, use multi-resolution and multi-feature fusion to improve the tracking precision, they sacrifice algorithm speed and are not able to satisfy our real-time requirement. There are several Siamese methods based on Siamese-FC, for instance, SA-Siam [36] uses two Siamese-FC networks to extract semantic feature and appearance feature, respectively. The fusion of two branches can also improve the tracking accuracy with doubled computational load. However, even Siamese-FC still suffers from background interference. An example of false tracking is displayed in Figure 3. 

In this video image sequence, the man walks on the sidewalk and passes buildings on the roadside. Our mission is to track the man and use the ground truth in the first frame as the target template. Because the Siamese network tracker treats the background in the ground truth bounding box as the tracking object template as well, when the man walks away in frame 92, the tracker is not able to track the target successfully and tracks on the background instead. After analyzing this example, we consider that the background has significant features, such as sharp edges or even similarities with target, which may cause this kind of phenomenon, especially when targets are small compared to the bounding boxes.

In our system, targets at the beginning are small in most situations because of the long distance form the camera in the sky. Furthermore, most of the current applications for real-time tracking start with cropping an object bounding box manually and are not able to generate suitable bounding boxes as ground truths in public datasets. Under this condition, we need to make our tracker more intelligent to differentiate targets and background. Consequently, we propose a method to get shape-adaptive templates from targets and to track them with a fully convolutional Siamese network.

## 3. Results

Our main idea is to design a network to track the target according to a manually-selected object in real-time video. Unlike most current tracking methods, which start with a precise ground truth and run a high score in public datasets, our network needs to get the best performance in our real-time system. In this section, we will introduce our method in four parts: the contour detection part, the template extraction part, the feature matching part, and the training part.

### 3.1. Contour Detection Network Architecture

A VGG-16 network has great depth and great density (16 convolutional layers and stride-1 convolutional kernels); it is widely used in classification tasks because it is easy to train and converges fast. Therefore, we adopt the architecture of VGG-16 network to extract features. In order to detect edge, some modifications are made in our contour detection network. Firstly, all the fully connected layers are deleted. Secondly, the last pooling layer is deleted. Thirdly, the output is connected with a refinement module. We take the Conv1, Conv2, Conv3, Conv4, and Conv5 with max pooling in a VGG-16 network as our first part. During the convolution process, we choose very small convolution filters (3 × 3), because it is the smallest size that can capture left/right, up/down, and center motions. The convolution stride is fixed to 1 pixel and max pooling is performed over a 2 × 2 pixel window, with stride 2. [29] The configurations of the receptive fields and strides are summarized in Table 1.

**Figure 4:**
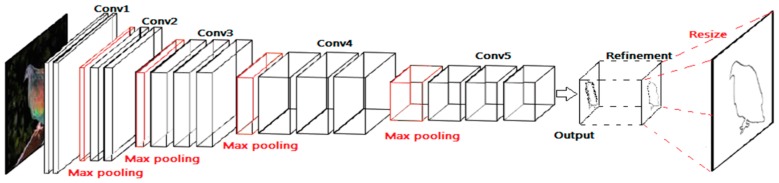
Architecture of the proposed contour detection network.

After analyzing the characteristics in different layers in a normal holistically-nested edge detection (HED) [37] network: the lower layers capture more spatial details, but lack sufficient semantic information; by contrast, the deeper layers encode richer semantic information, but spatial details are missing. Therefore, we learn from the idea of HED and choose to utilize the highest layers among the five side outputs. Especially in higher layers, the boundaries in edge maps suffer more from the problem of thick boundaries from the HED network. Consequently, the output needs to be refined and resized by up-pooling and transposed convolution/deconvolution to original size. All in all, the architecture of the proposed contour detection network is shown in Figure 4. 

The following is the comparison of the results of Canny, HED, and our network. 

**Figure 5:**
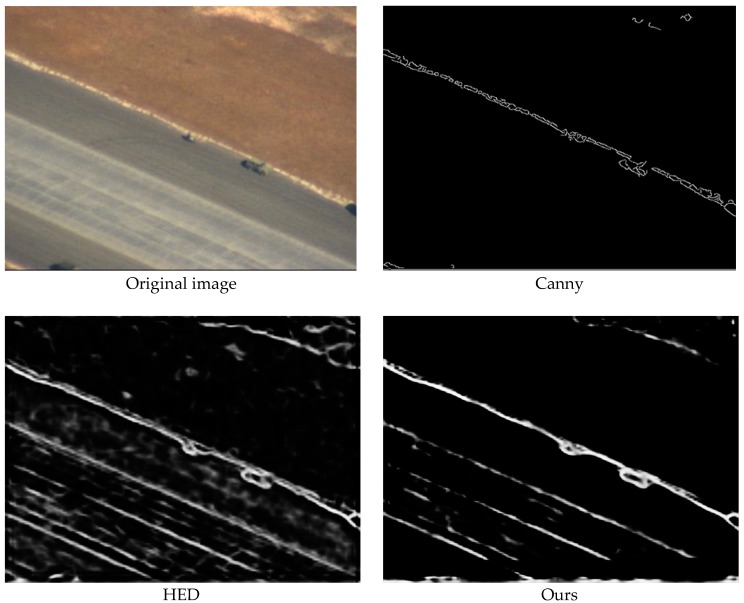
Results of different methods on an image taken by DARPA.

Figure 5 shows the results of different methods on a vehicle image taken by DARPA. Canny is an edge detection filter that simply detects edge information according to pixels which have the highest gradients in each area. HED has five side outputs (HED-dsn1, HED-dsn2, HED-dsn3, HED-dsn4, and HED-dsn5): the first three lower layers capture more spatial detail, while the last two higher layers capture semantic information; and the final output (HED-fuse) simply averages independent predictions from all side output layers without exploring the hierarchical feature representations of the convolutional network. [38] As a result, the final output of HED has more useless background information and interference from object texture. Our method, based on the highest layer which has low resolution and rich semantic information, has the best performance for detecting contours among the three methods. More details about the contour detection network were discussed in our previous paper [39].

### 3.2. Template Extraction Module

**Figure 6:**
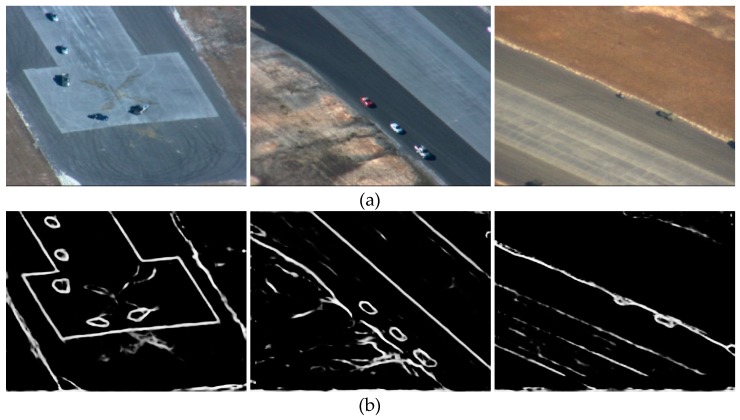
Results of our contour detection method: (**a**) Original image; (**b**) Contour.

The output of our contour detection network is a binary image with edge information. From the edge map, we need to firstly extract the object contour and generate a mask with the contour information. Then, the object can be discriminated from the background. Instead of traditional bounding box inputs, our template extraction module can generate an active template with object contour information. The advantages of an active template compared with bounding boxes have been discussed in Section 2.2. As a result, the accuracy of our contour detection module is significant and can improve the performance of the entire system. We have tested our contour detection network on vehicle images taken from the air at Eglin Air Base during the DARPA VIVID program. Figure 6 shows the results of our contour detection method on vehicle images taken by DARPA. 

**Figure 7:**
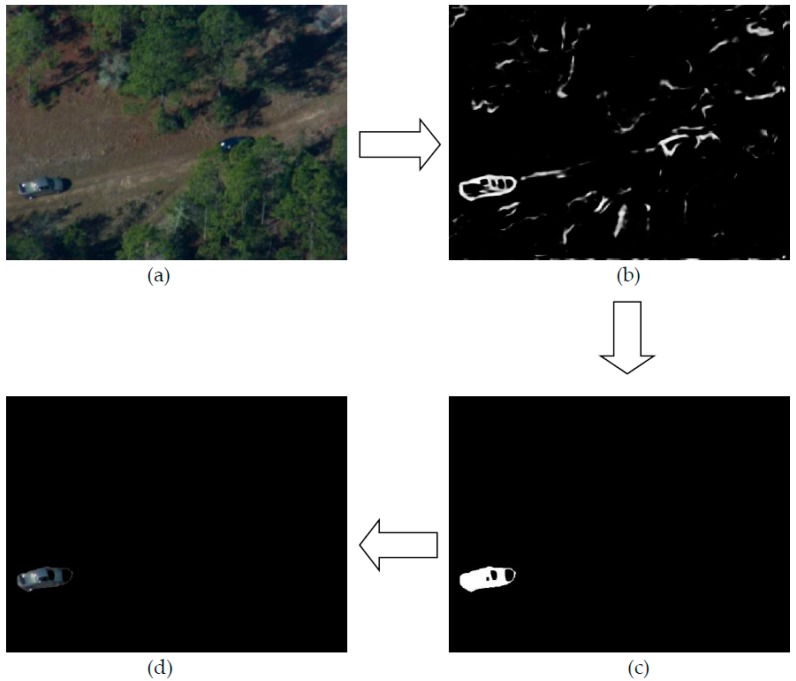
Sketch of our template extraction module: (**a)** Original image; (**b**) Contour; (**c**) Mask: (**d**) Target.

In order to realize the function of “start with a point”, we firstly utilize the flood fill method in OpenCV to generate a connected region to represent the position of our selected target from the contour map. Next, we can further produce a mask of the object based on the previous stage. The mask sets the background information as zero and the selected object is segmented from the original image and its features can be extracted as a target template without background information. A brief diagrammatic sketch is shown in Figure 7.

**Figure 8:**
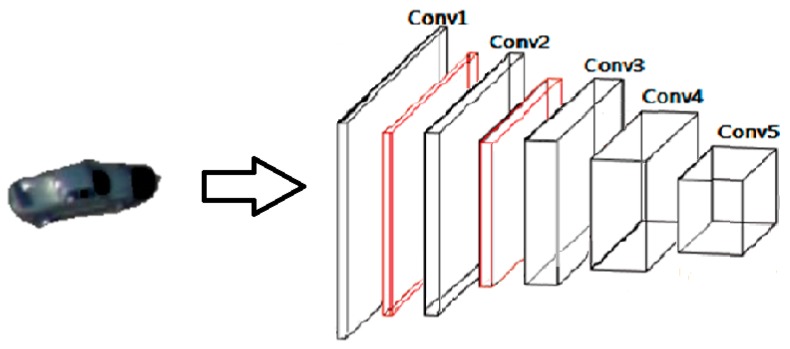
The schematic diagram of our feature extraction network’s structure: black boxes denote convolutional layers and red boxes denote max pooling layers.

To formulate out Siamese network, we revise and adopt the convolutional stage of the network designed by Krizhevsky et al. [30] for both the target template and the search region; the last three fully connected layers are deleted because our mission is to extract features rather than classification. The schematic diagram of our feature extraction network structure is shown in Figure 8.

The kernel size of the first convolutional layer is 11 × 11 × 3 with a stride of 4 pixels. After response-normalizing and pooling the output of the first layer, the second convolutional layer takes it as input and filters by kernels of size 5 × 5 × 48. The third convolutional layer is connected to the normalized and pooled output of the second layer with kernel size of 3 × 3 × 256. The following two convolutional layers are connected without any intervening pooling or normalization layers with kernel sizes of 3 × 3 × 192 and 3 × 3 × 192, respectively. The configurations of the receptive fields and strides are summarized in Table 2.

After extracting features of the target, we need to find the target within search region in the next frame. Here, we adopt the technique of stereo matching [40] to match score maps. The details will be illustrated in the next part.

### 3.3. Fully Convolutional Siamese Network Architecture

For most tracking methods, such as GOTURN and SiamFC, the first step is to scale and crop the input target image in the case of a lack of target feature information. During this operation, the added margin for context has benefits when the target is moving partially outside the bounding box, but at the same time, may cause the target’s reduction in size and introduces more background information. GOTURN utilizes the regression result in the last frame and updates the template in each frame, while SiamFC generates a template from the ground truth in the first frame throughout. In our approach, we do not update the template because this template is manually selected and has no non-rigid transformation: manually selected means it is a relatively good template compared with those in other frames, and vehicles are usually rigid objects. As a result, the template is fixed once selected by a human in the first frame, while the center of each search region is the position of target in previous frame. Now, the only remaining problem is finding the target in the search region. We finally utilize a correlation operation to compute the feature similarity between the template and the search region and generate a similarity heat map. The highest score in the heat map denotes the location of the target’s center in the next frame. To sum up, this loop from frame to frame achieves the function of tracking in our system.

**Figure 9:**
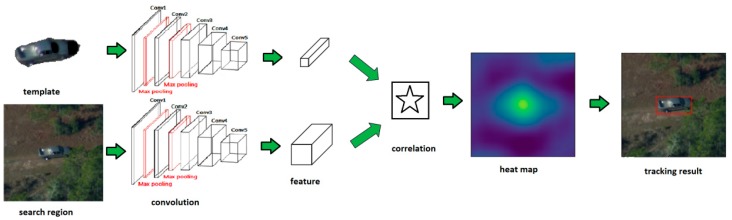
Main framework of our fully convolutional Siamese network.

Figure 9 shows the architecture of the fully convolutional Siamese tracking network: Firstly, the fully convolutional network takes the template and the search region as input separately and outputs features for both of them. Then, the similarity of the template’s and the search region’s features is computed through correlation and a heat map is generated. Finally, the object is located in the search region according to the heat map.

### 3.4. Details of Training End-To-End

In our approach, we pick template and detection patches as sample pairs from two frames with a random interval of the same video in ImageNet Large Scale Visual Recognition Challenge (ILSVRC) [41]. ILSVRC is a challenge to classify 30 different classes of animals and vehicles and detect these objects from videos. Therein, the vehicle videos with ground truth of location can help with our end-to-end training in the vehicle tracking system. The learning rate is set to $10^{-6}$ and the batch size is set to 8. We train our fully Siamese network end-to-end using straightforward Stochastic Gradient Descent (SGD) to minimize the loss function. The logistic loss is defined as:

(1) $$l(y,v)=\log(1+e^{-yv})$$

where y∈{+1,−1}, y∈{+1,−1} is the ground-truth label and *v* is the score which denotes the similarity of each exemplar candidate pair. Because each candidate pair consists of an exemplar image and a search region, this generates a score map D→R. We define the mean of the individual losses as the score map loss *L*:

(2) $$L(y,v)=\frac{1}{\left|D\right|}\sum_{u\in D}l(y\left[u\right],v\left[u\right])$$

where y[u]∈{+1,−1} for each u∈D is required as a true label.

## 4. Experiments and Results

Our segmentation and tracking networks were implemented based on TensorFlow framework with OpenCV 2.7 for Python. We firstly ran our tracking network on public datasets OTB100 and compared with other methods. In this section, we selected 11 vehicle videos in datasets: BlurCar1, BlurCar2, BlurCar3, BlurCar4, Car1, Car2, Car24, Car4, CarDark, CarScale, and Suv. After following the instruction of OTB100 benchmark, the precision and success plots of OPE were plotted separately. Then, we tested our system on real-world scenarios with images taken by a camera on an unmanned aerial vehicle. This set of experiments was performed by comparing the performance of the SiamFC with fixed bounding boxes as input and our fully convolutional Siamese network with shape-adaptive templates. The resolution of this set of images was 640 × 480. From 5 series of videos, we chose 2500 images constituting our datasets for testing. All the above experiments were tested on a server with a TITAN X GPU and a 3.5 GHz CPU.

### 4.1. Results on OTB100

SiamFC achieved state-of-the-art performance of object tracking of the OTB100 datasets. Because of the differences between the OTB100 challenge and our requirements, here we added a new group label for vehicle videos and modified the fore-end of our tracking network for testing. To be specific, we utilized the center point of the ground-truth bounding box as our “starting point”. 

We tested the one-pass evaluation (OPE) metric, which is accordance with our system. The precision plots of OPE and Success plots of OPE are shown in Figure 9.

**Figure 10:**
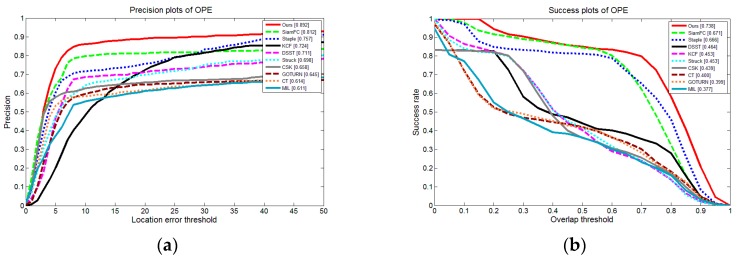
Results of the top 10 trackers of OTB100 vehicle videos: (**a**) Distance precision based on one-pass evaluation (OPE); (**b**) Success rate based on OPE.

From Figure 10, we can see that our proposed shape-adaptive template method achieves the best performance among these trackers. Figure 10a,b shows the distance precision and the overlap precision in OPE. In this set of experiments, our method obtained a 0.892 success rate at a 20-pixel threshold in the distance precision and an AUC score of 0.738 for overlap precision. Our method achieves the best performance for both of them: for distance precision, our method improves the performance by 9.9% when compared with the second-ranked tracker SiamFC.; for overlap precision, our method outperforms SiamFC by 10.0%.

### 4.2. Results on DARPA VIVID Datasets

#### 4.2.1. Qualitative Evaluation

As mentioned before, in our program, the images were taken from distance by a camera on a fast-moving unmanned aerial vehicle. This resulted in low resolution and small targets. We used similar datasets collected at Eglin Air Base from airborne sensor platforms by DARPA. The following two sets of images are from datasets taken by DARPA and are the results of the fully convolutional Siamese tracking network with different input methods, including fixed bounding boxes and shape-adaptive templates. 

**Figure 11:**
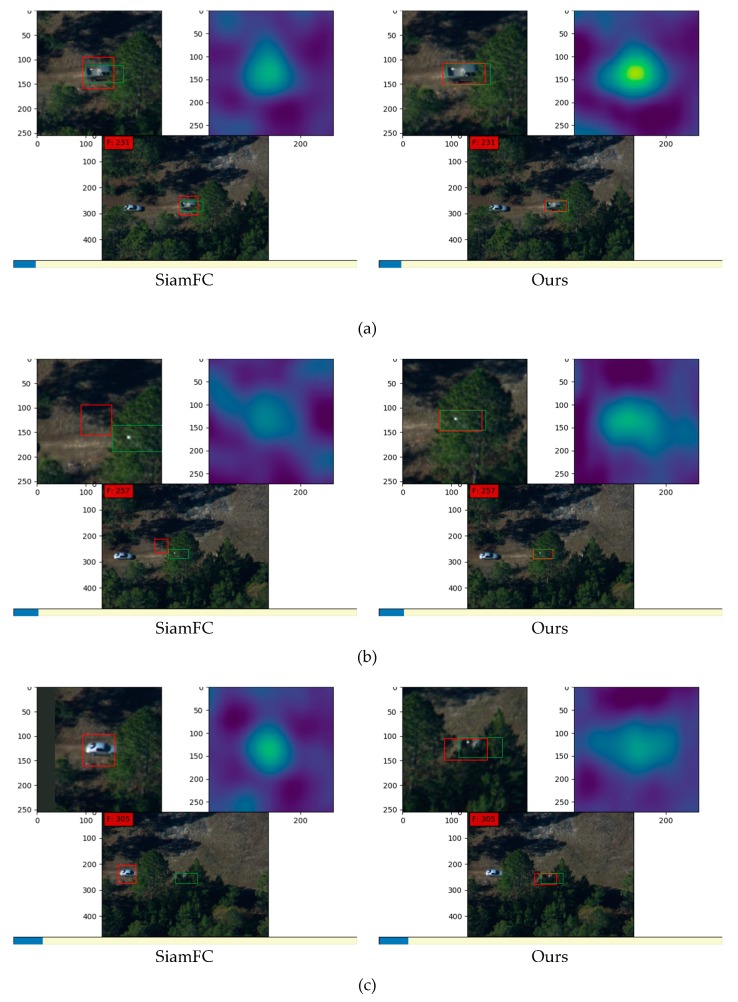

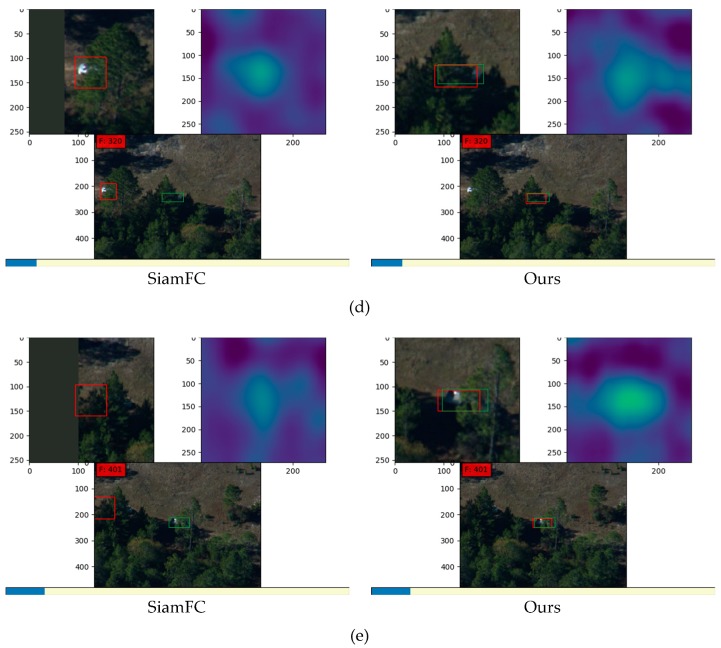
Results on images in datasets taken by DARPA: (**a**–**e**) are five groups of comparison between the tracking results of SiamFC and our method. In each group, the left images are results from SiamFC and right images are results from our method. For each method, the image at the bottom is the tracking result in the origin frame, the image at the top left corner is the partially enlarged detail of the tracking result in the current frame, and the image at the top right corner is the corresponding score map that denotes the similarity. All the green boxes denote the ground truth and all the red boxes denote the tracking results.

Figure 11 shows results on one sequence of video images in datasets taken by DARPA. SiamFC utilizes traditional bounding boxes as input and our method utilizes shape-adaptive templates which were generated by the segmentation masks. In Figure 11a, although both methods successfully track the target, the heat maps show that our method has much higher response according to the similarity. This results from our shape-adaptive templates that reduce the interference of background information. Besides, our method achieves a more precise result with higher overlap rate because of the successful learning of the target’s semantic information from the contour segmentation stage. In Figure 11b, the target is partially concealed by trees. Under this condition, SiamFC fails to track the object and obtains the highest similarity score at the wrong position that is similar to the background in the template bounding box. In contrast, our method has higher robustness with occlusions and tracks successfully. In Figure 11c, SiamFC false tracks another car following behind the target pick-up truck, while our method performs much better. In Figure 11d,e, SiamFC has interference from occlusion and loses the target, while our method tracks correctly from the beginning to the end.

#### 4.2.2. Quantitative Evaluation

In order to make the result a clearer and more visual, we calibrated and recorded the deviation of results in both SiamFC and our method.

It is worth noting that although our shape-adaptive template method has a much higher overlap score due to the template segmentation step, for fairness, we only compared the center precision score for success rate at a threshold of 20 pixels. This is because, in our system, center precision was the most important feature and the experimental results may vary according to different sizes of input bounding boxes. For accordance, we compared different methods with the calibrated ground truth every 10 frames and drew the figures below for 5 video sequences.

**Figure 12:**
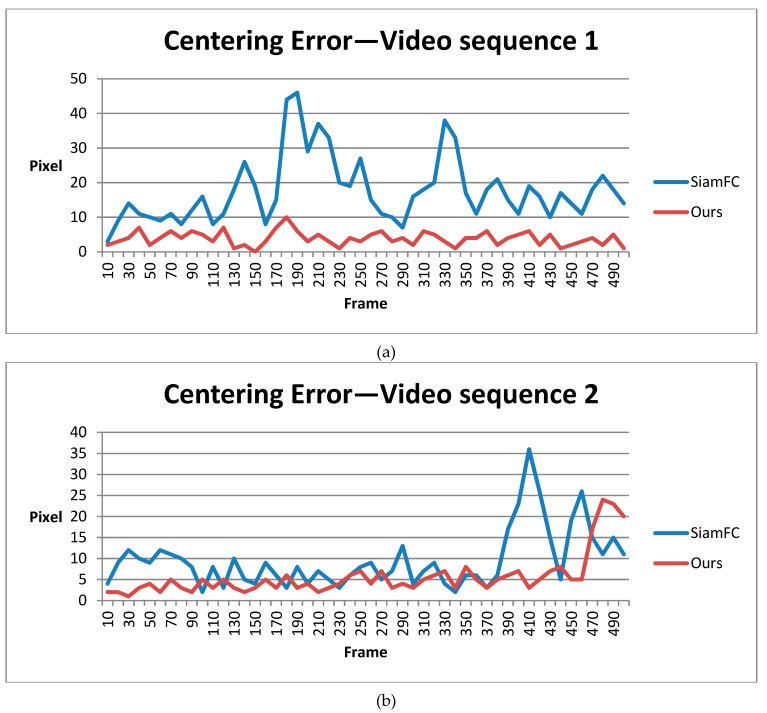

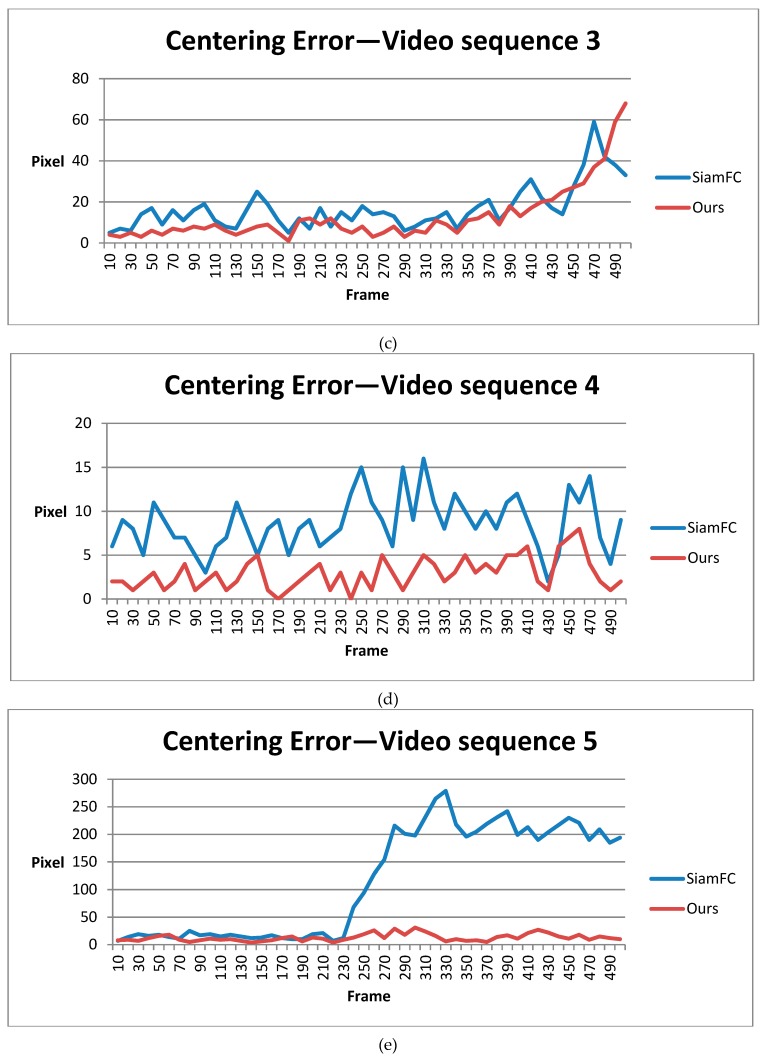

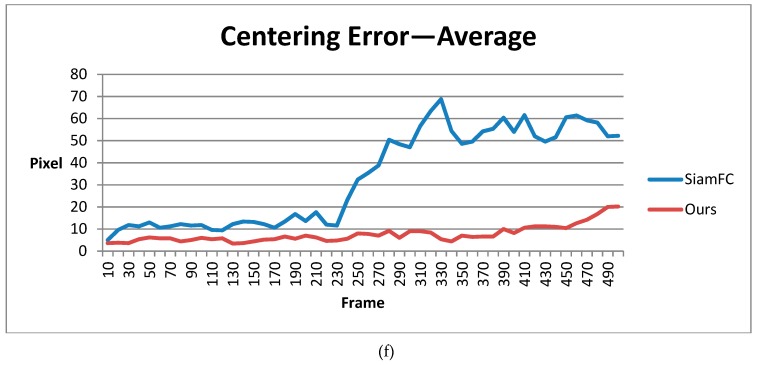
Statistical results of SiamFC and our method in DARPA VIVID datasets: (**a**–**e**) are centering errors in five different video sequences; (**f**) is the average centering error.

Figure 12 shows the statistical results for 5 video sequences and the average of the centering error in pixels. From video sequence 1 to 4, the target sizes are about 20 to 40 pixels, which is very small. In most frames, our method has a centering error of less than 10 pixels. This deviation is tolerable as long as the detected target’s center is still the real target’s position. Images in Figure 9 are from video sequence 5. In this video sequence, the target size is about 100 pixels, which is larger than those in the previous four. Our method’s centering error is mostly below 20 pixels, while SiamFC suffered from false tracking. Overall, our method has smaller centering error and outperforms SiamFC regarding images taken at Eglin Air Base by DARPA.

## 5. Conclusions

In this paper, we firstly analyzed the properties and drawbacks of current tracking methods regarding our requirements. After that, we proposed and implemented our “start with a point” tracking system: modifying and combining the contour detection network and the fully-convolutional Siamese tracking network to track in real time using shape-adaptive templates which has advantages over the traditional bounding boxes start-up. Experimental results show that our method has the best performance on vehicle videos in both OTB100 datasets and DARPA datasets.

## Figures and Tables

**Table 1:** The receptive field and stride size in our contour detection network. (RF is short for receptive fields, C is short for convolution, and P is short for pooling).

Layer	C1_2	P1	C2_2	P2	C3_3	P3	C4_3	P4	C5_3
RF size	5	6	14	16	40	44	92	100	196
stride	1	2	2	4	4	8	8	16	16

**Table 2:** The receptive field and stride size in our Siamese network (RF is short for receptive fields, C is short for convolution and P is short for pooling).

layer	C1	P1	C2	P2	C3	C4	C5
RF size	11	15	31	39	71	103	135
stride	4	8	8	16	16	16	16
